# Maternal angiotensinogen (*AGT*) haplotypes, fetal renin (*REN*) haplotypes and risk of preeclampsia; estimation of gene-gene interaction from family-triad data

**DOI:** 10.1186/1471-2350-11-90

**Published:** 2010-06-10

**Authors:** Hege K Vefring, Line Wee, Astanand Jugessur, Håkon K Gjessing, Stein T Nilsen, Rolv T Lie

**Affiliations:** 1 Department of Medical Biochemistry, Stavanger University Hospital, Stavanger, Norway; 2 Division of Epidemiology, Norwegian Institute of Public Health, Oslo, Norway; 3 Craniofacial Research, Musculoskeletal Disorders, Murdoch Childrens Research Institute, Royal Children's Hospital, Parkville, Australia; 4 Department of Obstetrics and Gynecology, Stavanger University Hospital, Stavanger, Norway; 5 Department of Public Health and Primary Health Care, University of Bergen, Bergen, Norway

## Abstract

**Background:**

Preeclampsia is a debilitating disorder affecting approximately 3% of pregnant women in the Western world. Although inconclusive, current evidence suggests that the renin-angiotensin system may be involved in hypertension. Therefore, our objective was to determine whether the genes for placental renin (*REN*) and maternal angiotensinogen (*AGT*) interact to influence the risk of preeclampsia.

**Methods:**

Three haplotype-tagging SNPs (htSNPs) covering *REN *(rs5705, rs1464818, and rs3795575) and another three covering *AGT *(rs2148582, rs2478545 and rs943580) were genotyped in 99 mother-father-child triads of preeclampsia pregnancies. We estimated relative risks (RR) conferred by maternal *AGT *and fetal *REN *haplotypes using HAPLIN, a statistical software designed to detect multi-marker transmission distortion among triads. To assess a combined effect of maternal *AGT *and fetal *REN *haplotypes, the preeclamptic triads were first stratified by presence/absence of maternal *AGT *haplotype C-T-A and tested for an effect of fetal *REN *across these strata.

**Results:**

We found evidence that mothers carrying the most frequent *AGT *haplotype, C-T-A, had a reduced risk of preeclampsia (RR of 0.4, 95% CI = 0.2-0.8 for heterozygotes and 0.6, 95% CI = 0.2-1.5 for homozygotes). Mothers homozygous for *AGT *haplotypes t-c-g and C-c-g appeared to have a higher risk, but only the former was statistically significant. We found only weak evidence of an overall effect of fetal *REN *haplotypes and no support for our hypothesis that an effect of *REN *depended on whether the mother carried the C-T-A haplotype of *AGT *(p = 0.33).

**Conclusion:**

Our findings indicate that the mother's *AGT *haplotypes affect her risk for developing preeclampsia. However, this risk is not influenced by fetal *REN *haplotypes.

## Background

Preeclampsia is a heritable, complex and serious disorder affecting approximately 3% of pregnant women in Western populations [1]. The underlying disease mechanisms are still unclear, but placental factors have been suggested to operate via the maternal circulation in causing endothelial dysfunction [2]. Maternal symptoms include hypertension and proteinuria. Early in a preeclamptic pregnancy, the spiral arteries are abnormally remodeled, leading to deficient placental perfusion. For preeclampsia to occur, however, reduced placental perfusion may have to interact with maternal factors [3].

Recent epidemiological studies have demonstrated that the risk of preeclampsia is determined not only by maternal predisposition, but also by a fetal contribution inherited from the father. Pregnant women whose partner had fathered a preeclamptic pregnancy with another woman were nearly twice as likely to have preeclampsia [4]. In studies of the risk of preeclampsia across generations, partners of men who were born to a preeclamptic pregnancy also had increased risk of developing preeclampsia [5,6]. Despite evidence for both maternal and fetal genetic contributions to the risk of preeclampsia, most published reports are based on the case-control design that does not distinguish between maternal and fetal gene-effects. Moreover, most of these studies have focused entirely on maternal susceptibility genes [7,8].

Preeclampsia could be associated with a specific combination of maternal and fetal alleles of the same or possibly different genes. Indeed, there are several examples of maternal-fetal gene-gene interactions in the literature. For example, placental factors (determined by fetal genes) have been suggested to raise maternal blood pressure, whereas maternal factors (determined by maternal genes) may counteract this rise in blood pressure [9]. Blood pressure is regulated by the renin-angiotensinogen system (RAS) through a cascade of AGT cleavage, generating the physiologically active angiotensin II. REN catalyzes the rate-limiting step of this cascade, the conversion of angiotensinogen to angiotensin I, and thus determines the activity of the system [10]. It is not known, however, whether the RAS system is involved in the basic etiology of preeclampsia or simply in regulating the maternal response to the condition.

An interaction of fetal *REN *with maternal *AGT *has been demonstrated in animal models. When transgenic female mice expressing human *AGT *were mated with transgenic males expressing human *REN*, the pregnant females displayed a transient elevation of blood pressure in late pregnancy due to secretion of placental human *REN *into the maternal circulation [11]. Similar results were obtained from experiments in a transgenic rat model of preeclampsia [12].

Given the strong link between *REN *and *AGT*, we aimed to assess whether there is a combined effect of feto-placental *REN *and maternal *AGT *on risk of preeclampsia in humans. We selected haplotype-tagging SNPs (htSNPs) covering each of the two genes and explored their combined association with preeclampsia in a clinically well-described set of Norwegian family triads from preeclamptic pregnancies. We estimated the effects of maternal *AGT *haplotypes, fetal *REN *haplotypes, and the combined effect of maternal *AGT *and fetal *REN *haplotypes.

## Methods

### Study participants

From January 1993 to December 1995, umbilical cord blood was collected from 8615 neonates delivered at the Stavanger University Hospital in Norway as part of a larger prospective study on risk factors associated with sudden infant death syndrome (SIDS). Women who fulfilled the diagnostic criteria for preeclampsia (n = 129), their partner and their affected child were invited to participate in the study [13]. Of these, one-hundred and two family triads were recruited in the study. Mothers and fathers donated peripheral blood post partum and gave written informed consent on behalf of themselves and their child. The study was approved by the Regional Committee for Ethics and the Norwegian Data Inspectorate.

### Genetic analysis

Genotype data from a 40 kb-long region covering *REN *(GeneID: 5972) and *AGT *(GeneID: 183), respectively, were downloaded from the genome browser of the International HapMap Consortium http://www.hapmap.org and imported into Haploview 3.0 [14] for computation of linkage disequilibrium (LD) statistics, identification and evaluation of htSNPs. Only SNPs with minor allele frequencies > 0.1 and haplotype blocks in LD with r^2 ^> 0.8 were imported. NCBI assembly 34 of the human genome and data release 14 were used. The exact locations of the htSNPs were determined using the BLAT utility at the UCSC Genome Bioinformatics site http://www.genome.ucsc.edu.

DNA was extracted from heparin blood using the QIAmp Blood Mini Kit (Qiagen, GmbH, Hilden, Germany). The selected htSNPs for *REN *[rs5705 (A/c; lowercase denotes the minor allele and uppercase the major allele), rs1464816 (G/t), rs3795575 (C/t)) and *AGT *(rs2148582 (C/t), rs2478545 (c/T), rs943580 (A/g)] were genotyped by the SNaPshot ddNTP primer extension method (Applied Biosystems, CA, USA). PCR primers and SNP extension primers were synthesized by MWG Biotech http://www.the-mwg.com and are listed in Table 1. Threeplex PCR reactions were performed for each patient as follows: three pairs of PCR primers were amplified simultaneously in 12 μl reactions containing 10 mM Tris HCl, pH 8.3, 50 mM KCl, 2.5 mM MgCl_2_, 0.2 mM deoxynucleotide triphosphates, 1 M betain, 0.05 U/μl AmpliTaq Gold DNA polymerase (Applied Biosystems) and 0.1 μg DNA. For each htSNP, forward and reverse primers were added to a final concentration of 0.2 μM, except for the rs3795575 primers that were added to a final concentration of 0.5 μM. *REN *threeplex amplifications were initiated by activation of the DNA polymerase for 9 min at 95°C, followed by 26 cycles of denaturation at 95°C for 30 sec, annealing at 60°C for 30 sec and polymerization at 72°C for 45 sec in a Master cycler XP (Eppendorf AG, Hamburg, Germany). For the *AGT *threeplex reactions, forward and reverse primers were added to a concentration of 0.5 μM, amplifications were performed without betain, and annealing was conducted at 58°C. The resulting PCR products (5 μl) were cleaned with EXOSAP-IT (2 μl) (Amersham Biosciences AB, Uppsala, Sweden) following the SNaPshot reaction.

**Table 1:** PCR primers and SNP extension primers

SNP	Primer sequence (5'- 3')	SNaPshot product size
*REN*		
rs5705	REN-F: CAAGAGAATGCCCTCAATCC	
	REN-R: CAAGCACTCACGTCCATGTAG	
	SNP-F: AGTGGAGCCAACCCATGAAGAGGCTGAC	29 bp
		
rs1464816	REN-F: TCCTTGGTTGGAGTCTGGTC	
	REN-R: GCTTTTCTTTTGCTGCTTGG	
	SNP-F: **AAAAA**CACAGAGTGTGTGCGTGCAGGGTTGAGG	34 bp
		
rs3795575	REN-F: AAGAAGCCAAAGAGGGAAGG	
	REN-R: GAAAGAGATGTCGGGGAGTG	
	SNP-F: **AAAAAAAAAA**ACCAGCGCAGGACTCCTTGTCTGCTGAGA	40 bp
		
*AGT*		
rs943580	AGT-F: CACAGAAAACAGCGGGAGAA	
	AGT-R: GGCTTGGAAGTTGCTCGTAG	
	SNP-F: AGGAGTATAAAGTTGCCAAC	21 bp
		
rs2148582	AGT-F: CTGCCGTTGTTCTGGGTACT	
	AGT-R: CACAAGCCCTGCTATTCCTC	
	SNP-F: **AAAAA**AGTTACATCTGAGAGAGACAAG	28 bp
		
rs2478545	AGT-F: ACCACGACAACCTCCTTGAG	
	AGT-R: GACCCATTTCAGATGCCACT	
	SNP-F: **AAAAAAAAAA**GCCTGCTGTCCCTAGGAGAAGTG	34 bp

SNP extension primers were designed to target DNA sequences next to their polymorphic sites, with a 5'-tail differing in lengths of 20 to 39 nucleotides (Table 1). The single-base extension reaction (10 μl) contained 3 μl of SNaPshot Multiplex kit (Applied Biosystems), 1 μM pooled SNP extension primers and 0.5 μl *REN *or *AGT *threeplex PCR product. Samples were amplified for 25 cycles with 10 sec of denaturation at 96°C, 5 sec of annealing at 50°C and 30 sec of polymerization at 60°C. The reaction products were treated with Shrimp Alkaline Phosphatase (Amersham Biosciences) for 60 min at 30°C followed by 30 min at 75°C. The resulting *AGT *or *REN *threeplex SNaPshot products (0.5 μl) were added to 20 μl of Hi-Di™ Formamide containing 0.2% Liz 120 size standard (Applied Biosystems) and loaded on an ABI PRISM^® ^310 instrument (Applied Biosystems) to separate the products. Genotyping was performed in duplicate. For genotype determination, the results were analyzed using the GeneMapper software, v3.0 (Applied Biosystems). Genotypes of the *REN *and the *AGT *htSNP were validated by direct cycle sequencing of 10 randomly selected family triads.

### Statistical analysis

One important purpose of this study was to demonstrate an approach for studying maternal-fetal gene-gene interactions. We used the HAPLIN statistical software package http://www.uib.no/smis/gjessing/genetics/software/haplin; [15]) to reconstruct haplotypes from htSNP genotype data and to estimate the relative risk associated with a single or double dose of each haplotype among the mother-father-child triads. An attractive feature of HAPLIN is that relative risk estimates for maternal and fetal alleles/haplotypes are unconfounded. Since our purpose was to estimate the joint effect of maternal *AGT *haplotypes and fetal *REN *haplotypes, we first identified maternal *AGT *haplotypes that could be associated with risk. Next, we stratified the preeclampsia triads by presence/absence of these maternal *AGT *risk-haplotypes to see whether the effect of fetal *REN *haplotypes differed significantly across strata of maternal *AGT *haplotype. A likelihood-based test was performed to test for such a difference.

## Results

**Figure 1:**
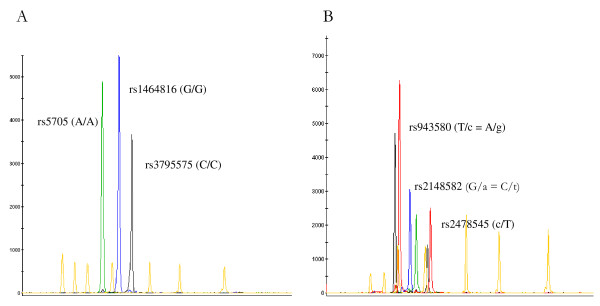
**Example of a typical *REN *(A) and *AGT *(B) threeplex reaction **A. The SNaPshot result of a homozygous carrier of the most common *REN *haplotype, A-G-C (SNP order: rs5705, rs1464816 and rs3795575). B. The SNaPshot result of a patient carrying both the *AGT *haplotype C-T-A and haplotype t-c-g (SNP order: rs2148582, rs2478545 and rs943580). Lowercase denotes the minor allele at the locus and uppercase the major allele.

One-hundred and two mother-father-child triads of preeclamptic pregnancies were genotyped for three htSNPs covering *REN *[rs5705 (A/c), rs1464816 (G/t), rs3795575 (C/t); Figure 1A] and three additional htSNPs covering *AGT *[rs2148582 (C/t), rs2478545 (c/T), rs943580 (A/g); Figure 1B]. Three family triads were excluded from the analyses because of inconsistent genotypes between the mother, father and child. Duplicate analysis of the *AGT *and *REN *threeplex reactions was performed on the remaining 99 family triads; no inconsistencies in genotype were observed. Further, none of the htSNPs showed any statistically significant deviation from Hardy-Weinberg equilibrium.

### *REN *and *AGT *haplotypes

With three diallelic htSNPs, there are eight possible haplotypes for each gene. We estimated, separately, the effects of carrying one and two copies of *REN *haplotypes among mothers and their offspring (a total of four sets of analyses). This was repeated for maternal and fetal *AGT *haplotypes. Haplotype frequencies, RR estimates with 95% confidence intervals (CI) and the corresponding p-values are presented in Tables 2 and 3. *REN *and *AGT *haplotypes with a frequency of less than 5% were excluded from the analyses because of the difficulty in obtaining valid RR estimates for rare haplotypes. Among *AGT *haplotypes, C-T-A was the most common (58.3%), while C-c-g and c-G-t had frequencies of 20.9% and 20.4%, respectively (Table 2A). Among *REN *haplotypes, A-G-C was the most prevalent (59.5%), followed by A-t-C and c-G-t, with frequencies of 31.6% and 8.5%, respectively (Table 2B).

**Table 2:** Relative risk estimates for *REN *(A) and *AGT *(B) haplotypes

A: Relative risk estimates (95% CI; p-value) for maternal *AGT *haplotypes (n = 87 family triads)			
***AGT *haplotype**	**Frequency (%)**	**Single dose**	**Double dose**

**C-T-A**	58.3	**0.39 (0.18-0.84); 0.02**	0.60 (0.24-1.51); 0.28
C-c-g	20.9	1.42 (0.65-3.12); 0.38	3.00 (0.93-9.93); 0.07
**t-c-g**	20.4	1.46 (0.66-3.20); 0.35	**4.63 (1.46-14.7); 0.01**

**B: Relative risk estimates (95% CI; p-value) for fetal *REN *haplotypes (n = 90 family triads)**			

***REN *haplotype**	**Frequency (%)**	**Single dose**	**Double dose**

**A-G-C**	59.5	0.63 (0.27-1.46); 0.27	**0.36 (0.13-0.98); 0.05**
A-t-C	31.6	0.86 (0.37-1.95); 0.72	0.40 (0.12-1.39); 0.15
c-G-t	8.5	1.45 (0.66-3.19); 0.35	Not estimable

SNP order for *AGT*: rs218582 (C/t), rs2478545 (c/T) and rs943580 (A/g)

SNP order for *REN*: rs5705 (A/c), rs1464816 (G/t) and rs3795575 (C/t)

### Maternal effects

There was a significant protective effect (RR = 0.4; 95% CI = 0.2-0.8; p = 0.02) among mothers heterozygous for the most frequent *AGT *haplotype, C-T-A (Table 2A). Among mothers homozygous for C-T-A, however, the protective effect was weaker and did not reach statistical significance (RR = 0.6; 95% CI = 0.2-1.5). Still, the estimated effects are in the same direction and indicate that presence of the C-T-A haplotype may be protective. As a result, we assumed a dominant effect for C-T-A in subsequent analyses. A risk of preeclampsia was also seen among mothers carrying the other two *AGT *haplotypes. Notably, mothers homozygous for the *AGT *haplotypes t-c-g and C-c-g had a RR of 4.6 (95% CI = 1.5-14.7, p = 0.01) and 3.0 (95% CI = 0.9-9.9, p = 0.07), respectively, with only the former being nominally significant. For heterozygous carriers of the same two *AGT *haplotypes, the corresponding RR estimates were 1.5 (95% CI = 0.7-3.2, p = 0.35) for t-c-g and 1.4 (95% CI = 0.7-3.1, p = 0.38) for C-c-g. Overall, the mother's *REN *haplotypes did not affect her risk of developing preeclampsia (data not shown).

### Fetal effects

Our data showed evidence of a protective effect on the risk of preeclampsia among children who were homozygous for the *REN *haplotype A-G-C (RR = 0.4; 95% CI = 0.1-1.0; p = 0.05) (Table 2B). In contrast, the child's *REN *haplotypes A-t-C and c-G-t did not affect the mother's risk of preeclampsia, and neither did the child's *AGT *haplotypes C-T-A, C-c-g or t-c-g (data not shown).

### *REN *and *AGT *interaction

To assess interaction between *REN *and *AGT *haplotypes, we fitted models with fetal *REN *haplotype effects into strata of families defined by maternal *AGT *haplotypes. One stratum consisted of mothers carrying the apparently protective C-T-A haplotype (57 family triads) (Table 3A), while the other stratum consisted of mothers who did not carry the C-T-A haplotype (27 family triads) (Table 3B). There was little evidence of an effect of *REN *in either of these strata. When we compared this stratified model with an overall model using a likelihood-based test, there was no evidence that fetal *REN *had a different effect based on presence/absence of the maternal *AGT *haplotype C-T-A (p = 0.33). We did not test for interaction between fetal *AGT *haplotypes and maternal *REN *haplotypes, as it was deemed implausible.

**Table 3:** Relative risk estimates for *REN *and *AGT *interactions:

A: Relative risk estimates (95% CI; p-value) for fetal *REN *haplotypes when mothers carry the protective *AGT *haplotype C-T-A (n = 57 family-triads)			
***REN *haplotype**	**Frequency (%)**	**Single dose**	**Double dose**

A-G-C	61.3	0.62 (0.22-1.71); 0.36	0.33 (0.09-1.19); 0.09
A-t-C	29.4	1.03 (0.38-2.82); 0.96	0.62 (0.14-2.79); 0.54
c-G-t	8.9	1.29 (0.50-3.25); 0.59	Not estimable

**B: Relative risk estimates (95% CI; p-value) for fetal *REN *haplotypes when mothers do not carry the protective *AGT *haplotype C-T-A (n = 27 family-triads)**			

***REN *haplotype**	**Frequency (%)**	**Single dose**	**Double dose**

A-G-C	55.2	1.53 (0.16-14.90); 0.71	1.0 (0.08-12.4); 0.99
A-t-C	38.0	2.40 (0.24-24.10); 0.46	0.89 (0.06-15.20); 0.93
c-G-t	5.8	0.60 (0.09-4.14); 0.60	Not estimable

SNP order for *REN*: rs5705 (A/c), rs1464816 (G/t) and rs3795575 (C/t)

## Discussion

We found a significant association between maternal *AGT *haplotypes and preeclampsia in our data. This is consistent with the results of a previous case-control study of French-Canadian Caucasians, where preeclamptic women carrying the *AGT *A-Met-Thr (G1035A-Thr174Met-Met235Thr) haplotype had a 2.1 fold (p = 0.0008) increased risk of disease compared to control subjects [16]. In contrast, a larger British study - also known as the Genetics of Preeclampsia Consortium (GOPEC) - failed to replicate an association of maternal *AGT *haplotype with preeclampsia after analyzing 398 maternal (grandmother-grandfather-mother) triads and 536 fetal (mother-father-child) triads [17].

Except for a borderline-significant double-dose effect of one of the haplotypes, fetal *REN *haplotypes did not appear to influence the mother's risk of preeclampsia. A large-scale genetic association study of 394 preeclamptic women and their 324 offspring from Chile [18] found no evidence of association between fetal *REN *variants and preeclampsia.

Our hypothesis and analyses were based on findings from a previous mouse-model study in which strong evidence of a gene-gene interaction between *AGT *and *REN *was found [11]. Specifically, preeclampsia-like symptoms were observed when females carrying human *AGT *were mated with males carrying human *REN*, whereas pregnant mice derived from other mating combinations did not manifest preeclamptic symptoms. This suggests that human *REN *produced by the paternal gene in the placenta may enter the maternal circulation and interact with maternal *AGT *to increase the expression of symptoms of preeclampsia [11]. Similar results have been obtained from experiments in rats [12]. Since there is no evidence that the *REN *gene is imprinted, we did not differentiate between paternally-inherited *REN *and maternally-inherited *REN *when estimating the effects of fetal *REN*. There was no evidence of interaction between maternal *AGT *and fetal *REN *among our 99 preeclamptic mother-father-child triads.

Given our limited sample size, we decided not to test all possible combinations of maternal *AGT *and fetal *REN *haplotypes. One hundred triads should give adequate power (80%) to detect a relative risk of 2.5 or higher conferred by either a maternal or a fetal allele [19,20]. Our study is under-powered to detect moderate gene-gene interaction effects; thus, we cannot rule out such interactions. There is, however, little evidence of strong interactions in our data.

Although *REN *is an attractive candidate gene for preeclampsia, only a few studies have investigated associations of *REN *with preeclampsia. For example, *REN *did not feature in GOPEC's list of candidate genes [17]; only *AGT*, angiotensin II receptor 1 (*AGTR1*), coagulation factor V (*F5*) and II (*F2*), methylene tetrahydropholate reductase (*MTHFR*), endothelial nitric oxide synthase (*NOS3*) and tumor necrosis factor alpha (*TNF*) were investigated. Despite recent reports supporting the hypothesis that both maternal and fetal genes may be involved in the development of preeclampsia, only a few studies have investigated fetal genetic contributions, and even fewer have used a study design that can distinguish between maternal and fetal gene-effects. Identification of joint maternal and fetal risk factors for preeclampsia is important for better defining risk and risk outcomes.

In conclusion, we examined the possibility of an interaction between maternal *AGT *and fetal *REN *haplotypes by applying new statistical methodologies that can estimate these effects without confounding in a family-triad setting. We found an association between maternal *AGT *haplotypes and preeclampsia, and a weak association between fetal *REN *and preeclampsia. There was, however, no interaction between maternal *AGT *and fetal *REN *haplotypes. The mother-father-child triad approach is recommended in future genetic studies of preeclamptic risk factors since this design enables the separate estimation of maternal and fetal gene-effects as well as interaction of maternal and fetal genes.

## Competing interests

The authors declare that they have no competing interests

## Authors' contributions

RTL contributed to the conception and design of the study, carried out statistical analysis and drafted the manuscript. HKV selected htSNPs, designed and carried out SNaPshot analysis, performed DNA sequencing and drafted the manuscript. LW performed SNaPshot and sequence analysis. AJ selected htSNPs, provided input in the overall study design, and critically reviewed the manuscript. HKG wrote the HAPLIN program and duplicated the statistical analyses. STN participated in patient recruitment and validated the clinical information for the preeclampsia study. All authors read and approved the manuscript.

## Pre-publication history

The pre-publication history for this paper can be accessed here:

http://www.biomedcentral.com/1471-2350/11/90/prepub
